# What factors impact the social mobility of athletes retired from Liaoning Province professional sports teams? A qualitative research approach

**DOI:** 10.3389/fspor.2026.1943882

**Published:** 2026-10-19

**Authors:** Shuai Hao, Haotian Xing, Haibo Tian

**Affiliations:** 1 Department of Physical Education, Shaoxing University, Shaoxing, China; 2 Faculty for Physical Education, Zhejiang Yuexiu University, Shaoxing, China

**Keywords:** family background, mobility-related attractions, personal effort, relational society, retired athletes, social mobility, sport expertise

## Abstract

**Introduction:**

Approximately 3,000 to 6,000 Chinese athletes retire annually, and 90% of them must independently navigate their subsequent career paths. Against the background of this large-scale, self-directed career transition, it is critical to explore the underlying factors shaping retired athletes' post-retirement development. This study primarily investigates the key factors influencing the social mobility of athletes after retirement.

**Method:**

In-depth interviews were conducted with 20 retired athletes aged between 34 and 57 years old who had previously trained as players in Liaoning professional sports teams.

**Results:**

Five major themes emerged through a thematic analysis: (a) an unavoidable family background, (b) established sports expertise, (c) substantial personal efforts, (d) attractions shaping mobility choices, and (e) the balance of bureaucratic culture and relational society.

**Discussion:**

Given these contributing factors, retired athletes exhibit distinct variations in their social-mobility experiences. The findings of this study contribute to our understanding of the social mobility process of retired athletes and offer insights into the career mobility of these individuals.

## Introduction

As the share of retired athletes within the employed population grows, concerns regarding “social mobility” and strategies to improve retired athletes’ career development have attracted scholarly attention (1). Liaoning Province, as a traditional powerhouse of competitive sports in China, has cultivated a large number of elite athletes across summer sports, winter sports, and team sports, and shoulders important responsibilities for talent reserve and competitive performance for the development of national sports in China (2). Every year, a large number of athletes withdraw from professional training and competition due to age, injury, physical condition, and team adjustments, forming a special group that faces career transition and social re-adaptation (3). Retired athletes’ social mobility in terms of occupational status, economic income, social identity, and social capital directly reflects the effectiveness of athlete training, resettlement, and social security systems. It is also closely linked to regional sports talent stability, social equity, and the high-quality development of sports (4, 5).

Against the background of China's sports system reform, the transition from the traditional policy-driven resettlement model to a market-oriented and diversified employment model has brought both opportunities and challenges to retired athletes in Liaoning (6). On one hand, the expansion of the sports industry, the deepening of sports-education integration, and supportive local policies have broadened employment channels including coaching, physical education teaching, sports management, and entrepreneurship (7). On the other hand, long-term closed training, inadequate cultural education, narrow professional skill sets, chronic sports injuries, difficulties with identity adaptation, and constraints imposed by regional economic and industrial structures have led to clear differentiation in social mobility: a small number of elite athletes achieve upward mobility, while most ordinary retired athletes face barriers including difficult career transition, low occupational stability, and limited space for upward mobility (8, 9).

Existing domestic and foreign studies have explored the career transition, employment dilemma, and influencing factors of retired athletes from the perspectives of sociology, psychology, management, and economics (1, 10). However, most studies focus on national-level macro analysis or individual cases, and lack targeted qualitative research on Liaoning Province, which has a distinctive sports talent structure and regional context (2). In particular, the subjective experiences, practical constraints, and structural factors behind mobility disparities among Liaoning's retired athletes remain insufficiently explored through qualitative approaches including in-depth interviews and narrative analysis.

Therefore, this study adopts a qualitative research approach, taking retired athletes from Liaoning provincial professional sports teams as the research object, and uses in-depth interviews and thematic analysis to explore the key factors affecting their social mobility. Based on Bourdieu's capital theory and athlete career transition theory (4, 11), this paper constructs an analytical framework of individual, institutional, policy and regional contextual factors, aiming to answer the following core question: What individual endogenous and external structural factors jointly shape their social mobility trajectories? Theoretically, this study can enrich regional empirical research on the social mobility of Chinese retired athletes, and offer a qualitative perspective for understanding the interaction among individual capital, institutional arrangements and regional context. Practically, it can provide evidence-based references for Liaoning Province to adjust athlete resettlement policies, optimize career transition services and improve retired athletes’ social adaptation, and also offer reference for other provinces with similar sports talent backgrounds.

## Literature review

### Social mobility

Social mobility refers to the movement of individuals or social groups across different positions within the social stratification system, and is commonly categorized into intragenerational mobility and intergenerational mobility, as well as vertical (i.e., upward/downward) mobility and horizontal mobility (12). Classic sociological theories emphasize that mobility is jointly shaped by structural factors and individual agency. Wajdi et al. argued that industrialization and educational expansion boost the openness of stratification systems and expand mobility opportunities (13). Turner distinguished between sponsored mobility and contest mobility, offering a clear framework for analyzing how institutional selection mechanisms influence individual status attainment (14).

Bourdieu's capital theory has become a core analytical tool for mobility research (4). He conceptualizes capital as existing in four distinct forms: economic, cultural, social, and symbolic, and these forms can be converted into one another to jointly influence status attainment. Cultural capital (i.e., education, tastes, credentials), social capital (i.e., networks, trust, relational resources), and habitus jointly affect an individual's ability to move across different social positions (15). Recent studies further highlight the role of regional context, place attachment, and policy environment in shaping mobility outcomes, especially for marginalized or specialized groups (16, 17).

Within this study, social-mobility is conceptualised as intragenerational shifts in multidimensional social-stratification positions after athletes exit the provincial elite sport system. Mobility trajectories are assessed relative to each participant's baseline social position held during their elite athletic career. Given the qualitative design of this work, we do not assign numerical stratification scores or allocate individual participants to fixed discrete mobility categories. Instead, social-mobility is empirically inferred from four observable dimensions: occupational status, economic income, social identity, and changed access to social network resources (an outcome dimension of mobility, distinct from pre-existing social capital resources that shape mobility as an antecedent). Interpretive rules guiding group-level inference are as follows: relative to pre-retirement baseline, collective improvements across multiple dimensions suggest upward mobility; net deterioration across key dimensions indicates downward mobility; broadly comparable standing corresponds to horizontal mobility; inconsistent changes across dimensions constitute mixed multidimensional mobility.

### Competitive sport and social mobility

Elite sport has long been regarded as a potential channel for upward social mobility, particularly for individuals from disadvantaged backgrounds. Sport can compensate for deficits in family-origin capital and provide access to education, networks, and symbolic status (18). Existing research in Europe and North America shows that athletic achievement helps individuals accumulate cultural and social capital, thereby increasing their access to elite education, management positions, and public service roles (5, 11).

However, sport does not guarantee social mobility and may even reinforce existing inequality. Studies reveal that mobility opportunities are unevenly distributed: athletes with higher competitive levels, formal education, and supportive backgrounds are more likely to achieve upward mobility, while those with lower rankings, early retirement, or limited education often face stagnation or downward mobility (9, 19). Gender, ethnicity, and sport type further shape mobility outcomes: female athletes and those in less commercialized sports often encounter stronger mobility barriers (20, 21).

In the Chinese context, the state-run elite sports system forms a unique social mobility mechanism. On one hand, athletes gain institutional resources, honorary status, and resettlement opportunities from the system. On the other hand, long-term specialized training may weaken their general human capital. Moreover, the transition from policy-based resettlement to market-oriented employment has increased employment uncertainty, leading to significant stratification among retired athletes (2, 6).

### Influencing factors of social mobility among retired athletes

Existing systematic reviews and empirical qualitative studies have developed a multi-layered analytical framework covering individual endogenous factors, sports institutional factors, regional policy factors and market environmental factors. The interplay of these multiple factors collectively affects the direction, speed and upper limit of retired athletes’ social mobility, and each dimension contains complex internal mechanisms.

Individual endogenous factors are the core internal constraints shaping athletes’ capital conversion and occupational mobility, including human capital, psychological capital, athletic identity, and demographic characteristics. First, structural imbalance in human capital constitutes the fundamental barrier to mobility. Sport-specific human capital has low cross-industry applicability. Long-term closed training crowds out formal academic education, leaving most athletes deficient in general vocational competencies. Chronic athletic injuries further weaken labor capacity and raise medical costs; severely injured athletes actively narrow their job scope, limiting occupational choices (22). Second, psychological capital and athletic identity moderate the efficiency of capital conversion. Over-reliance on athletic identity causes post-retirement role confusion, low self-efficacy and career inaction. By contrast, athletes with strong psychological resilience and proactive career planning can effectively accumulate new capital to achieve upward mobility, and psychological capital plays a critical mediating role in shaping mobility outcomes (20, 23). Third, demographic traits create differentiated mobility thresholds. Most Liaoning athletes retire between the ages of 24–34, facing a structural age disadvantage in the labor market. Female athletes may encounter implicit recruitment barriers and bear heavier family care burdens. Additionally, a favorable family socioeconomic background provides auxiliary social capital for securing high-quality employment.

The institutional rules governing competitive sports determine athletes’ initial capital endowment, forming fundamental external institutional constraints for their post-retirement mobility. First, the separation between sports training and education results in insufficient institutionalized cultural capital. Unlike Western dual-career training models, Liaoning provincial teams prioritize competitive performance over cultural education. Adult education accessed after retirement receives little recognition in the labor market and cannot remedy the long-term discontinuity in athletes’ educational trajectories (24). Second, achievement-oriented differentiated resource allocation solidifies social mobility stratification. Institutional placement, medical security, subsidies and further education quotas are all tied directly to competition medals. Elite medal-winning athletes gain access to high-quality institutional resources, while ordinary grassroots athletes only receive meager one-time compensation and low-quality basic vocational training, which widens the mobility gap within the group (2). Third, imperfect pre-retirement transition support systems hinder athletes’ capital reconstruction. Teams typically only provide short-term, generalized vocational training, and lack personalized career planning, advanced skill courses and professional psychological counseling. The training content rarely covers qualifications for emerging sports-related posts. Furthermore, defective occupational injury security policies increase the risk of downward mobility for injured athletes (6).

Regional policies, the industrial landscape, and sociocultural environments act as external constraints limiting the capital conversion efficiency of retired athletes in Liaoning. In terms of policy, while local resettlement policies are already in place, they suffer from prominent implementation gaps. Ongoing public sector staffing cuts have reduced the number of government-allocated sports posts; athlete-oriented preferential policies are characterized by cumbersome procedures and narrow coverage, and only provincial elite athletes can access full policy benefits (6). As documented in prior research (2), Liaoning's economy is dominated by traditional heavy industry, with an underdeveloped modern sports sector. From participants’ accounts, this structural condition creates limited high-end market-based positions for retired athletes. The region lacks high-quality market-oriented positions for retired athletes, and most available roles are low-income grassroots fitness posts. Additionally, regional winter sports face prominent seasonal off-season employment barriers (2). In sociocultural terms, stereotypes held by the public and employers label athletes as having low academic and comprehensive abilities, giving rise to invisible employment discrimination. Furthermore, the insufficient economic vitality of Northeast China compresses the overall upward mobility space for all labor market participants.

### Research gaps

Existing literature has laid a solid foundation for relevant research, yet it still has three key limitations: 1) Most studies employ macro quantitative methods and lack in-depth qualitative exploration of subjective experiences; 2) Regional research on major sports provinces such as Liaoning remains scarce; 3) Few studies systematically integrate capital theory and career transition theory to explain the interactive mechanisms among individual, institutional, and regional factors. According to the major concept of capital theory (e.g., social capital) and career transition theory (e.g., career transition resources), the primary goal of this study is to identify multi-layered influencing factors that drive or constrain these observed social mobility.

## Method

Qualitative approach is suited to capture the complex mechanisms of retired athletes’ social mobility, as it explores retrospective narratives and reveals hidden structural barriers that large-scale quantitative surveys fail to fully capture. This study employed in-depth interviews as its qualitative research approach due to its versatile and flexible characteristics (25). This approach explores specific topics with a clear research focus, and has the following advantages: interaction between interviewers and respondents, flexibility for individualized expression, and the possibility of asking improvised follow-up questions (26, 27). These advantages are particularly well-suited for exploring unclear phenomena, which is exactly the case for the social mobility changes of retired athletes (28).

## Participants

Purposive sampling deliberately recruited retired athletes with divergent mobility experiences, maximizing information richness for exploring causal mechanisms rather than aiming for statistical representativeness. Recruitment combined key informant referral and snowball sampling. Potential participants were identified with assistance from former coaches and retired sport administrators of Liaoning provincial level sport teams, then contacted via telephone or email with study related information. Interviewees were also invited to refer other eligible participants. Eligibility criteria targeted participants with complete, unbuffered post-retirement social-mobility trajectories rooted in Liaoning's elite-sport system. These athletes previously trained as professionals at a Liaoning provincial team. The inclusion criteria for respondent selection were as follows: (a) aged 30 or older; (b) prior experience training as a player in a professional sports team; (c) retired for more than one year; (d) not an Olympic champion, and not a professional athlete pursuing further education after retirement. As shown in Table 1, a total of 20 respondents were purposively recruited for this study in accordance with the predefined inclusion criteria. Participants ranged in age from 34 to 57 years (average age = 40.7), and the sample was evenly split between male and female respondents. Half of the respondents are currently employed in Liaoning Province, while the other half work in other provinces. Most respondents hold an athlete grade of Level 1 or above, and the sample covers a variety of sports including football, gymnastics, and weightlifting. The vast majority of respondents currently hold occupations that differ from their original athletic careers.

**Table 1:** Sample basic situation statistics table.

NO.	Sport event	Gender	Age	Work place	Athlete class	Previous occupation	Current occupation
P1’	Athletics	M	43	Chengdu	International athlete	Assistant coach	University Teacher
P2’	Athletics	F	35	Guiyang	International athlete	University Teachers	University Teacher
P3’	Fencing	F	34	Beijing	First class athlete	Fencing Club Coach	Head of Fencing Club Coaching Department
P4’	Tennis	M	40	Shenzhen	Secondary athlete	Secondary School Teacher	Secondary School Teacher
P5’	Volleyball	F	47	Tianjin	Athlete	Unemployed	Secondary School Teacher
P6’	Rowing	M	39	Ningbo	Athlete	Shopping security; Courier	Farmer
P7’	Trampoline	F	40	Nantong	Athlete	Unemployed	University teacher
P8’	Football	F	57	Chongqing	Athlete	Foreign college coach	Professional sports team coach
P9’	Gymnastics	F	52	Shanghai	First class athlete	Professional sports team coach	Professional sports team coach
P10’	Hockey	F	35	Beijing	Athlete	Primary School Teachers	Professional sports team coach
P1	Track and field	M	40	Liaoning	Athlete	Taxi driver, Primary School Teachers	Police officer
P2	Gymnastics	F	41	Liaoning	International athlete	Coach	University Teacher
P3	Tennis	M	39	Liaoning	First class athlete	Tennis Club Coach	Professional sports team coach
P4	Athletics & Alpine skiing	M	37	Liaoning	International athlete	Small vendor	Government department staff
P5	Triathlon	M	37	Liaoning	First class athlete	Unemployed	Fire brigade officer
P6	Kayak	F	36	Liaoning	First class athlete	Health Club Manager, Mall Manager	Ordinary government officials
P7	Free sparring	M	46	Liaoning	Athlete	Underground Black Market Boxer	Corporate department manager
P8	Weightlifting	F	39	Liaoning	International athlete	Various odd jobs	Unemployed
P9	Speed skating	M	40	Liaoning	Secondary athlete	Car mechanic	Individual business
P10	Judo	M	37	Liaoning	Secondary athlete	Shopping mall salesperson;Fitness club trainer	Individual business

F, female; M, male; P represents employment in Liaoning Province, P’ represents working in other provinces.

## Data collection

Before formal interviews commenced, all participants received information about the study, including its research purpose and clear notification that participation was entirely voluntary. Written informed consent was obtained from each participant, who retained the right to withdraw from the interview at any time. Data collection was conducted through a combination of online and offline methods, using one-on-one, in-depth interviews with respondents. Each interview began with the question, “When did you start professional training in competitive sports?” It then proceeded to ask respondents to describe their experiences and challenges encountered during the transition from an active sporting life to retirement, exploring how they adapted to post-retirement life and what influence their career changes had after retirement. A sample interview question is as follows: “Please tell us about your work experience from your retirement to the present.” Individual interviews were conducted from May to October 2023, with durations ranging from 41 to 85 min. After obtaining written informed consent, the researchers recorded each complete interview using a smartphone voice recorder and transcribed the audio verbatim into MS Word documents immediately after each interview concluded.

## Data analysis

This study used open and axial coding as analytical operations within a thematic analysis framework (29). These coding procedures serve to systematically identify, compare, and cluster recurring meaning units from interview transcripts, rather than applying full grounded theory protocols. Drawing on Merriam's approach to inductive category construction, open axial coding guided our stepwise generation of empirical themes from raw interview material. During open coding, researchers repeatedly examined interview transcripts to extract inductive core concepts capturing retired athletes’ social mobility related experiences. Following iterative development and refinement of the initial codes, axial coding was used to cross-examine conceptual relationships; this clustering process produced the five empirically derived themes, which were then assessed for adequacy against interview data. Coding followed dual inductive deductive logic: codes were induced from participant narratives, whereas Bourdieu's capital theory and athlete career transition theory operated as sensitising interpretive lenses, not predefined coding categories that would constrain theme formation.

The adequacy of our 20-interview dataset is justified by the information power principle (30). The focused objective, purposive maximum variation sampling, established theoretical frameworks, rich in-depth interview data, and cross-case comparative analysis ensure sufficient relevant information to answer our core research question. Investigator triangulation (31, 32) was adopted to improve research reliability (33). The team comprised the first author and two researchers specializing in sociology of sport with qualitative experience. All three coded independently, then compared outputs in joint meetings. They discussed validity, mutual exclusivity, distinctiveness, and exhaustivity, reaching full consensus on final coding.

All interviews were conducted in Mandarin Chinese and transcribed verbatim in Chinese. Selected quotations from participants were translated into English by the first author. Translation quality was verified via back-translation: an independent bilingual researcher translated the English quotes back into Chinese (34). The two Chinese versions were then compared, and any semantic inconsistencies were discussed and revised to retain the original meaning intended by participants.

## Results

Our results are interpreted against the above qualitative operationalisation of social mobility. Rather than prioritising the classification of every participant into discrete mobility categories, the analysis focuses on unpacking key factors that shape intragenerational multidimensional social-stratification shifts after athletic retirement. Five major themes emerged from the research data (see Table 2): (a) an unavoidable family background, (b) established sports expertise, (c) substantial personal efforts, (d) attractions shaping mobility choices, and (e) the balance of bureaucratic culture and relational society.

**Table 2:** Mapping between themes and relevant theoretical concepts.

Inductively-derived empirical theme	Relevant theoretical concepts	Theoretical origin	Empirical illustration in this study
a. An unavoidable family background	Economic capital; social capital; habitus	Bourdieu (4)	Family financial resources, pre-existing kin-based social networks, family-shaped career values constitute antecedent capital resources shaping athletes’ post-retirement trajectories
b. Established sports expertise	Sport-specific embodied cultural capital; symbolic capital; transition resources	Bourdieu (4); Stambulova & Henriksen (11)	Competitive achievements and sport-related skills constitute resources and prestige; also tied to risks of rigid athletic-identity lock-in
c. Substantial personal efforts	Capital-conversion practices; individual transition resources and agency	Bourdieu (4); Stambulova & Henriksen (11)	Athletes’ self-study, skill-rebuilding and proactive strategies to transform existing capital into new social-stratification outcomes
d. Attractions shaping mobility choices	Contextual pull-push factors	Stambulova & Henriksen (11)	Regional economic benefits, city reputation and environmental conditions shape geographical and occupational mobility decision-making
e. The balance of bureaucratic culture and relational society	Social capital; *inductively-generated local contextual extension*	Bourdieu (4)	Formal bureaucratic rules and informal relational resources jointly enable or block capital conversion; a China-specific empirical extension of social-capital thinking

Themes were first induced inductively from interview transcripts. Theoretical concepts serve as sensitising interpretive lenses for these already-emerged themes, rather than pre-defining thematic categories prior to data analysis.

### An unavoidable family background

A prominent inherent factor highlighted in the interviews is the positive support of family background for retired athletes’ social mobility. When athletes make career transitions, they may face various job-seeking challenges due to insufficient social support. Social resources from one’ s family often serve as the primary source of support for an athlete, and typically, athletes who receive support from their parents and relatives during their transition find it easier to adapt to the changes they encounter. P1’ (M, 43) mentioned that his career transition went very smoothly, benefiting from his father's social resources:

*At a pivotal moment in my life, during a career transition, my father, drawing on his extensive industry experience and broad network of contacts, actively assisted me by making connections. He introduced me to senior figures in the industry, enabling me to access more firsthand information and job opportunities.*

P9’ (F, 52) provided an example of her teammate who used clan resources for career choice. She noted that the father of one of her teammates was a police officer who held significant authority. After her retirement, she took up a position as a security guard.

Abundant economic resources from family members can help retired athletes expand their employment opportunities. P6’ (M, 39) perceived that his family operates a highly profitable sea cucumber farming business. After he left his previous job, his family supported him in managing the family enterprise. P10 (M, 37) noted that he also encountered funding issues in the early stages of his business, and his parents provided significant assistance:

*Upon learning about the challenges I encountered while starting my business, my parents invested in me without hesitation. They sold a portion of their cultivated land and used the proceeds to open a women's fitness club.*

Family cultural background indeed influences the values that guide retired athletes during their career selection process. Families that prioritize educational inheritance are more inclined to have their children pursue careers in education, whereas families that emphasize economic gains may encourage their offspring to enter the business sector. This divergence in values can give rise to the “intergenerational transmission” of career paths. P1 (M, 40) was very excited about his current career due to his father's influence.

*My father is a university professor specializing in business planning. Influenced by him, I planned to pursue self-study before retiring, and eventually, I became a police officer.*

### Stablished sports expertise

Upon the conclusion of their athletic careers, athletes embark on a new chapter in life and assume new social roles. During the transition to these new roles after retirement, athletes may struggle to adapt to the corresponding behavioral requirements due to psychological and behavioral inertia. As noted by P6’ (M, 39): “After staying in the sports team for a long time, he was not adept at communicating with strangers. Many job interviews failed, and in the end, he could only work as a security guard in a shopping mall.” In addition, conflicts, oppositions, resistances, and contradictions frequently arise within a single role or between different roles. P4 (M, 37) describes the unique nature of professional athletes’ experiences:

*A professional sports career follows a different path from that of ordinary individuals. While their peers are engaged in schoolwork, athletes are in training. As peers build relationships with classmates and teachers, athletes forge bonds with teammates and coaches. While peers travel with their parents, athletes compete with their teams.*

If professional athletes continue to work in the sports industry or related fields after retirement, their specific sports background and competitive achievements can serve as valuable capital and advantages. As the primary criterion for evaluating Chinese athletes, sports achievements exert a significant influence on athletes’ acquisition of social status and identity recognition both before and after retirement. P1’(M, 43) stated that as a key member of the national team who has participated in the Olympic Games, it is perfectly reasonable for him to remain at the provincial team as a coach. Influenced by regional differences and varied social demands, sports events have a tangible impact on the social mobility of professional athletes after retirement. For example, P8 (F, 39) remarked:

*Sports events also have an impact after retirement. Some entertaining and easily accepted sports make it easier to find employment. Have you seen weightlifting at the fitness club? However, sports like tennis, basketball, and table tennis are very popular.*

The role transition of retired athletes involves adapting to and learning the behavioral norms and lifestyles necessary for engaging with society and securing new employment after retirement, as well as improving their overall quality of life. P7 (M, 46) notes that when the energy and perseverance gained during professional training are applied to one's current career, it is highly likely to create value and bring a decent income and social status. Additionally, they must continually cultivate new foundational qualities, such as fulfilling their professional role functions, internalizing the culture of their new job positions, and discarding or altering behavioral patterns that are incompatible with their new professional roles. P4 (M, 37) reflected that it is best for athletes to engage in their original sports or similar fields after retirement, and that they should not leave the sports industry; otherwise, all their years of hard work and dedication would be wasted.

### Substantial personal efforts

The Screening Hypothesis posits that education serves as a crucial indicator of an individual's capabilities. Employers use educational qualifications to assess job candidates’ skills and assign job roles based on their proven competencies. Typically, higher educational attainment corresponds to a higher salary offered by employers. Most respondents mentioned that a higher education background acts as the “key” to securing a stable job. Most athletes experience anxiety regarding their educational qualifications when contemplating post-retirement careers, as noted by P8’ (F, 57): “If an individual's educational level is comparatively low and does not meet the basic employment criteria for the majority of professions, there will be absolutely no prospect.” Clearly, through one's own relentless efforts, obtaining a higher degree helps people ascend to a higher social class. For example, P1’ (M, 43) described his story:

*When I was serving as an assistant coach for the provincial team, I saved all my salary and bonuses without enhancing my academic qualifications, solely to prepare for finding a job. Upon retirement, I was admitted to Beijing Sport University and had the honor of becoming a university professor.*

Outstanding athletic skills are not only the most fundamental labor skills of elite athletes, but also the most important manifestation of the value of their workforce. The cultivation and acquisition of motor skills are inherently scarce. The pyramid-shaped training model leads to the elimination of the vast majority of athletes, who often earn low incomes, are plagued by injuries, lack employment and survival skills, and face severe employment challenges. Most retired athletes recognize that transitioning from professional athletic roles to new positions requires continuous effort to learn new professional skills and adapt to different behavioral norms. For instance, P6’ (M, 39) stated, “I taught myself the relevant knowledge about fruit preservation and transportation, and then registered an online store to start selling the fruits from my own home.” P2’ (F, 35) also indicated her positive experience with her job:

*After retiring, she spent all her retirement benefits on studying traditional Chinese medicine and massage, obtained the qualification of a masseuse, and smoothly became a university teacher.*

### Attractions shaping mobility choices

This theme captures context-related pull-factors (i.e., pre-existing contextual attributes) that draw athletes toward specific geographic locations or occupational options. Importantly, these attractions constitute antecedent conditions for mobility-related decision-making, rather than representing a distinct dimension or outcome of social mobility itself. Geographical relocation or occupational change are potential behavioural consequences triggered by such attractions; Retired athletes’ geographical mobility arises from the joint contextual effects generated by their original locations and target destinations. Relevant pull attributes of destination regions that attract retired athletes encompass city-brand reputation, economic benefits, as well as natural and cultural environments.

In the perception of the Chinese public, the “city label” is an important pull factor for geographical mobility. For Chinese people, city labels and regional names represent unique regional characteristics, and also shape people's sense of identity. These markers can satisfy individuals’ desire for the positive status associated with geographical mobility, and may even foster a sense of comparative advantage. Many interviewees indicated that eastern coastal cities hold greater appeal for retired athletes. P9’ (F, 52) described what attracts her to work in Shanghai: “Shanghai, a municipality directly administered by the Central Government, has consistently been the most advanced region in China over the past several centuries, embodying state-of-the-art technology and a thriving economy.”

From a practical perspective, economic income constitutes a fundamental prerequisite for meeting survival needs in socioeconomic contexts, and represents a core consideration for retired athletes when seeking employment and pursuing social mobility. P9’ (F, 52) stated, “As a coach, the economy in South China is more dynamic, offering more opportunities and generating higher income, which naturally makes these clubs more attractive to me.” A high income is one of the key reasons why professional players choose their career path. An interviewee who had successfully transitioned to work as a secondary school teacher explained as follows:

*The school is located in the Shenzhen Development Zone. This position presents a highly attractive opportunity for me. The salary is very high, and they have also solved the problems of housing and transportation for me. (P4’, M, 40)*

Retired athletes must establish a new identity to earn a living, operate in new settings, and “reinvent” themselves after retirement. When asked which environmental factors could attract retired athletes to pursue social mobility, P6’ (M, 39) stated: “This project necessitates the availability of lakes and rivers, along with quality boats, equipment, and facilities. These should all be accessible on-site. Additionally, the air quality in this location is exceptionally good, rendering it an ideal venue for athletes to train.” Furthermore, another respondent noted that convenient transportation, well-developed facilities, and a rich cultural environment are also important environmental factors that attract retired athletes to choose this career (P10’, F, 35).

### The balance of bureaucratic culture and relational society

According to the interview accounts collected in this study, several participants described discretionary power related phenomena within Liaoning's sport sector institutions, as perceived by these respondents. It is important to emphasise that these are participants’ subjective perceptions and lived experiences rather than independently verified objective systemic conditions. From the researchers’ interpretive standpoint, these individual narratives collectively suggest that such perceived institutional dynamics may constrain institutional mobility pathways for retired athletes within the contexts experienced by our sample. For example, P3’ (F, 34) said: “Upon starting work, I realized there is a gap between the outward presentation and actual reality of teacher recruitment information. These announcements seem pre-determined, with specific details only released after the whole process is completed.” Similarly, P8’ (F, 57) recalled that two individuals without relevant professional backgrounds were appointed as assistant coaches for the women's football team before a major competition. In her view, these individuals contributed little to team preparation and appeared primarily motivated by competition results and prize money. She reported that collective appeals and negotiations did not result in any change to the arrangement.

Against the backdrop of China's efforts to build a “service-oriented government”, an increasing number of public officials have adopted a service-oriented mindset, moving away from bureaucratic behavior. For example, P2’ (F, 35) described this sense of service consciousness as follows: “Upon learning about her situation, the department head promptly called to invite her for an interview at the college and even booked a flight ticket for her.” P1’ (M, 43) spoke highly of the service awareness recruitment managers demonstrated in solving problems during his job selection process. He mentioned: “To alleviate any potential concerns I might have had, they specially arranged a position for my wife as a university teacher. Additionally, they implemented a “one-on-one” support policy for me when introducing me as a high-level coach candidate.”

Social relationships are stable relational structures between social actors, encompassing both strong and weak interpersonal ties. Existing research has found that strong social relationships play a dominant role in career mobility within Chinese society. In this study, respondents reported that robust social connections, such as those between superiors and subordinates, among teammates, and between relatives, can facilitate their social mobility. As P2 (F, 41) noted, Chinese people hold a high level of trust in acquaintances and friends, and in many cases, a friend's recommendation can be even more influential than an individual's actual abilities. P7’ (F, 40) also indicated the influence of social relationships on her employment:

*Given the nature of their profession, my parents were acquainted with numerous high-ranking government officials in the northeast China. Consequently, before I completed my university studies, a prestigious institution offered me a position as both a professional teacher and a gymnastics coach.*

In this study, significant others refer to individuals who play a crucial role in the social mobility of retired athletes. While the types of significant others identified in the interview data are diverse, their common feature is that they assist the social mobility of retired athletes by utilizing their various resources and social connections. Significant others also exert influence through social relationships, power status, or professional reputation. P1 (M, 40) stated that his inspirational coach had become the principal of a sports school, and after P1 retired, this coach hired him to serve as a coach at the school. Likewise, P4 (M, 37) made the following comment:

*The team leader arranged for my personnel file to be transferred to the Liaoning Sports Bureau through the documents issued by the National Sports Administration and the Snow and Ice Sports Management Center.*

## Discussion

Drawing on in-depth interviews data collected from 20 retired athletes and adopting thematic qualitative analysis, this study systematically explores the multi-dimensional influencing factors and formation mechanism of social mobility among retired athletes in Liaoning Province. By integrating Bourdieu's capital theory and athlete career transition theory, this research reveals the chained interaction effect of individual endogenous, institutional, regional policy, and socioeconomic environmental factors on athletes’ intragenerational social mobility. The core findings are discussed and compared with existing literature as follows.

### Endogenous individual factors dominate capital conversion and mobility outcomes

Consistent with previous empirical studies (8, 22), this study suggests that individual endogenous factors act as the core internal constraint on retired athletes’ social mobility. Our interview data further provides micro-level subjective experiential evidence that complements prior quantitative conclusions.

First, human capital deficiencies, including singularity of sport-specific skills, insufficient formal education, and chronic occupational injuries, remain the fundamental barrier to cross-industry and upward mobility. Most interviewed athletes reported weak competitiveness in the general labor market, which stems from long-term closed boarding training crowding out time for systematic cultural learning. Extending beyond existing quantitative results (35), this study finds that individual effort can effectively compensate for inherent human capital disadvantages: several respondents achieved career advancement by acquiring vocational qualification certificates and higher academic degrees through self-study, which suggests the screening hypothesis of educational attainment in social mobility research.

Second, this study highlights the critical role of family background and relational social capital, which were underemphasized in prior national-level studies. Thematic analysis of the unavoidable impact of family background indicates that pre-existing family economic, cultural, and relational capital form differentiated mobility thresholds for Liaoning retired athletes. Athletes with advantaged family resources gain priority access to employment information, job referrals, and entrepreneurial funding support, forming clear intergenerational transmission of career advantages (36). This conclusion echoes Bourdieu's argument that social capital and family habitus explain individual status attainment (15), and explains why identical institutional training resources lead to polarized mobility outcomes among peer athletes.

Third, psychological quality, athletic identity, and personal initiative significantly influence capital conversion efficiency (37). Respondents with proactive career planning and strong environmental adaptability can transform the perseverance and willpower cultivated in competitive sports into workplace advantages. By contrast, athletes who over-rely on athletic identity and hold negative career cognition fall into long-term career stagnation, which is consistent with the psychological regulation mechanism concluded by Demulier et al. (20). Notably, this research finds that personal effort can offset partial disadvantages of low initial capital endowment, breaking the absolute constraint of innate individual conditions on mobility trajectories.

### Sports institutional system solidifies initial capital stratification

This study suggests that the Chinese centralized competitive sports training system shapes athletes’ initial capital endowment, which forms the fundamental institutional foundation for subsequent mobility differentiation (2).

First, the long-standing separation of sports and education in Liaoning's provincial professional teams has created structural deficiencies in athletes’ institutionalized cultural capital. Different from the dual-career synchronous training models adopted in Europe and North America (38), Liaoning's performance-oriented training mechanism sacrifices athletes’ opportunities for formal education. Meanwhile, low-value adult education cannot remedy the educational disadvantage that athletes face in the labor market.

Second, the medal-linked differentiated resource allocation mechanism solidifies the hierarchical mobility gap between elite and grassroots athletes (39). Interview data shows that world-class and provincial elite medal winners can access targeted public institution placement and special policy subsidies, while ordinary athletes only receive one-time resettlement compensation paired with low-value short-term vocational training. This indicates the existence of sponsored mobility within the Chinese sports system: institutional resource distribution depends on symbolic capital (competition medals) rather than individuals’ comprehensive quality.

Third, according to participants’ lived accounts, an incomplete pre-retirement transition support system represents a commonly perceived institutional shortcoming within Liaoning's sports management. Most teams only provide standardized short-term vocational training, which lacks personalized planning and psychological counseling. This leaves athletes unable to complete systematic capital reconstruction before exiting the elite sports system (6).

### Regional context restricts external capital conversion efficiency

Unlike the findings of general nationwide studies, this study identifies unique regional constraints specifically tied to the socioeconomic context of Liaoning and Northeast China.

First, it is implementation deviation, rather than a lack of relevant policies, that hinders the high-quality employment of local athletes. Although Liaoning has introduced a complete set of resettlement policies for retired athletes, participants’ narratives further illustrate how these documented gaps are subjectively experienced at the individual level: the tightening of public sector staffing quotas, cumbersome application procedures, and tiered policy privileges have resulted in unequal benefit distribution: only elite athletes at the provincial level can fully access policy support, while grassroots athletes at the municipal and county levels are largely excluded from benefits.

Second, as documented in prior regional research (2), Liaoning's industrial structure constitutes a documented contextual bottleneck. The local economy is dominated by traditional heavy industry, and its modern sports sector remains under-developed. From participants’ accounts, this structural context translates into limited high-end positions in sports management, operation, and brokerage. Most market-based positions available to retired athletes are perceived as low paying grassroots fitness instructor roles. Participants also reported prominent seasonal off-season employment barriers within the regional winter sports sector, a contextual difference noted in comparison with southern coastal sports strong provinces (2).

Third, consistent with broader regional economic observations, Northeast China's limited economic vitality constrains general upward mobility space for labour market participants. From participants’ perspectives, persistent employer stereotypes regarding athletes’ academic capacity create perceived invisible employment related barriers, which, in their experience, limit the market conversion of athletes’ symbolic capital (5). Participants also perceived that the region's generally low wage ceiling constrains maximum achievable income for retired athletes, irrespective of individual personal resources.

### Interactive mechanism of multi-dimensional factors and regional relational characteristics

Interview results indicate that individual, institutional, and regional environmental factors do not operate independently. Instead, they form a chained multi-factor restriction mechanism for social mobility. Institutional rules suggest initial capital endowment, individual endogenous conditions reflect capital conversion ability, and regional policy and industrial environment influence the channels and efficiency of such conversion. The superposition of multi-dimensional disadvantages suggests a pattern of horizontal or downward mobility across most ordinary retired athletes in our sample; by contrast, elite athletes with abundant symbolic capital show evidence consistent with rapid upward mobility at the group level (40).

Most importantly, this study identifies two localized characteristic mechanisms unique to Liaoning Province. On the one hand, participants’ subjective narratives reflect that bureaucratic intervention and relation-based societal culture may disrupt fair institutional mobility channels. As reported by interviewees, administrative power from some sports departments interferes with public recruitment and internal placement assessments, undermining the fair competition-based mobility opportunities for grassroots athletes. On the other hand, regional urban reputations and economic returns create clear incentives for cross-regional mobility. Most competitive interviewees tend to relocate to eastern coastal first-tier cities with superior economic conditions and comprehensive industrial support, which further drains Liaoning's local sports talent resources.

### Research theoretical and practical implications

Theoretically, by integrating Bourdieu's capital theory and athlete career-transition theory, this study addresses three key research gaps in the existing literature. First, it reveals the micro-level action logic of multi-factor interaction based on athletes’ real career transition experiences, rather than merely identifying simple variable correlations verified through quantitative research. Second, it supplements targeted qualitative research focusing on typical northern sports powerhouse provinces. Third, it integrates capital theory and athlete career transition theory to construct a comprehensive multi-layered analytical framework for the social mobility of retired athletes.

Practically, the findings of this study provide targeted optimization suggestions for Liaoning's sports administrative departments. First, reform the current segregated sports and education training system to accumulate early-stage cultural capital for adolescent athletes. Second, build hierarchical and personalized pre-retirement transition service systems for elite and grassroots athletes respectively. Third, address documented gaps in policy implementation (6) and respond to participants’ reported experiences of perceived administrative influence and perceived employment-related barriers. Fourth, accelerate the cultivation of the local modern sports industry to expand high-quality market employment channels for retired athletes.

### Limitations and future prospects

This study has several notable limitations. First, it adopts small-scale purposive sampling, which does not support statistical generalization to the broader population. Second, its cross-sectional design fails to capture long-term dynamic changes in mobility among retired athletes. Third, this qualitative research cannot quantify the weight of multi-dimensional influencing factors, and overlooks group differences across different sport types. Fourth, snowball sampling may under-represent socially isolated athletes. Self-reports carry recall and self-selection bias; interviewer effects cannot be ruled out. Missing employer or institutional perspectives and independent verification may lose linguistic nuances.

Accordingly, future research should adopt mixed methods to quantify the contribution of each influencing factor, and conduct longitudinal tracking and cross-group comparative studies. From a practical perspective, targeted policy intervention trials are recommended to develop localized employment support schemes for retired athletes in Liaoning. Future research could use multi-party interviews, archival evidence, and cross-linguistic quality control to support targeted athlete policies.

## Conclusions

This qualitative study surveyed 20 retired professional athletes in Liaoning and identified five core factors of their post-retirement social mobility: family background, sports expertise, personal efforts, attractors shaping mobility choices, and the balance between bureaucratic and relational social culture. On the whole, social mobility among these athletes is shaped by the chained interaction of individual endogenous, sports institutional and regional socioeconomic factors. Among them, individual conditions serve as the core internal driving force, institutional arrangements shape athletes’ initial capital endowment. Consistent with prior regional context research (2), Liaoning faces documented structural limitations within its modern sports sector. Meanwhile, participants’ accounts point to relational culture related challenges as key perceived external constraints reported within our interview sample.

This study indicates the applicability of Bourdieu’s capital theory in athlete career research and provides empirical reference for optimizing the employment support system for retired athletes in Liaoning.

## Data Availability

The original contributions presented in the study are included in the article/Supplementary Material, further inquiries can be directed to the corresponding author.
